# Improving the HIV response for transgender populations: evidence to inform action

**DOI:** 10.1002/jia2.25993

**Published:** 2022-10-12

**Authors:** Tonia Poteat, Nittaya Phanuphak, Beatriz Grinsztejn, Sari L. Reisner

**Affiliations:** ^1^ Department of Social Medicine University of North Carolina School of Medicine Chapel Hill North Carolina USA; ^2^ Institute of HIV Research and Innovation Bangkok Thailand; ^3^ Evandro Chagas National institute of Infectious Diseases‐Fundação Oswaldo Cruz (FIOCRUZ) Rio de Janeiro Brazil; ^4^ The Fenway Institute Fenway Health Boston Massachusetts USA; ^5^ Division of Endocrinology, Diabetes and Hypertension Brigham and Women's Hospital Boston Massachusetts USA; ^6^ Department of Epidemiology Harvard T.H. Chan School of Public Health Boston Massachusetts USA

**Keywords:** HIV testing, HIV prevention, HIV treatment, HIV continuum, differentiated service delivery, HIV pre‐exposure prophylaxis

Our knowledge about HIV among transgender populations has substantially improved since the *Journal of the International AIDS Society* (JIAS) published its first special issue about HIV in transgender populations in 2016 [1]. A PubMed search indicates that more than 1000 peer‐reviewed manuscripts focused on HIV among transgender people have been published in the past 5 years. While this increased volume of research is heartening, the findings indicate transgender people remain disproportionately burdened by HIV globally. A recent meta‐analysis of studies published between 2000 and 2019 found an overall HIV prevalence of 19.9% for trans feminine individuals and 2.6% for trans masculine individuals with odds ratios of 66 and 7, respectively, compared to all individuals ages 15 years and older [2]. While data on the HIV prevention and care continuum among transgender people are limited, existing studies indicate lower engagement in HIV testing, pre‐exposure prophylaxis (PrEP) uptake, persistence and adherence, as well as antiretroviral therapy (ART) uptake and adherence [3]. These appalling inequities in HIV prevalence, prevention and care are driven by socio‐structural factors rooted in stigma and discrimination [4].

In keeping with the most recent World AIDS Day call to “end inequalities” as well as the AIDS 2022, the 24th International AIDS Conference theme, “re‐engage and follow the science,” we dedicated this special issue to research that fills important gaps in science and provides data that can be used to end inequities in HIV prevention and care for transgender people. We called for abstracts that fill gaps in knowledge about trans masculine people, gender non‐binary people, transgender people in sub‐Saharan Africa, as well as data on community‐based and clinical interventions. Manuscripts by transgender authors were encouraged. We were excited to receive more than 70 abstracts. Through a careful process of editorial and peer review, we selected 13 quality manuscripts that contribute to the evidence base for advancing equity for transgender people in the HIV response. This issue includes research from countries across the globe, including rare data focused on transgender people in sub‐Saharan Africa; studies inclusive of and/or focused specifically on transgender men; and programmatic data that provide practical insights into potentially effective strategies to engage transgender people in HIV prevention and care. We are delighted that more than half of the manuscripts in this special issue are led or co‐authored by transgender and nonbinary people.

We include two manuscripts that address some of the extensive gaps in HIV research focused specifically on the experiences and needs of transgender men. Radix et al. provide one of the largest clinical data sets on HIV prevalence and sexual risk among transgender men in the United States. Data from this relatively diverse sample of 577 transgender men found that White participants were less likely to be tested for HIV, Black participants were more likely to be living with laboratory‐confirmed HIV and HIV prevalence was highest for transgender men who reported having only cisgender male partners [5]. In multivariable analyses, race was no longer significantly associated with HIV status; however, participants with only cisgender male partners had a statistical odds of living with HIV that was 10‐fold higher than those without a cisgender male partner. The authors underscore the need to collect better data on sexual practices among transgender men, particularly those who have sex with cisgender men, in order to include them in HIV prevention efforts for men who have sex with men (MSM) and to develop culturally and anatomically appropriate educational materials for them.

Appenroth and colleagues directly compare self‐reported sexual health outcomes of 122 transgender MSM with more than 22,000 cisgender MSM in Germany [6]. In their study, transgender MSM were less likely to self‐report living with HIV but were also less likely to report receiving an HIV test result, suggesting that some transgender MSM may be living with HIV but are unaware because they have not been tested. Transgender MSM were less likely than cisgender MSM to have ever talked to a provider about PrEP, representing a missed opportunity for HIV prevention education for this group. Compared with cisgender MSM, transgender MSM had a higher odds of reporting sexual unhappiness, having sex that was less safe than they wanted, having an income too low to live comfortably and having negative mental health symptoms. These psychosocial and structural differences may play a role in HIV vulnerability for transgender men, deserving further exploration in future research. Taken together, the Radix and Appenroth studies begin to paint a picture in which transgender MSM may face elevated HIV vulnerability compared to heterosexual transgender men yet face higher barriers to HIV testing and PrEP uptake than cisgender MSM. More research, as well as interventions tailored to the specific needs of transgender MSM, are clearly needed.

Four manuscripts expand the evidence base on the uptake of HIV testing and prevention among transgender populations. Lacombe‐Duncan et al. examine the prevalence and associated factors for HIV testing among 539 transgender and gender non‐binary people in the Midwestern United States [7]. More than a quarter (26.2%) of participants had never been tested for HIV. The highest proportion to never have an HIV test were non‐binary people assigned female at birth (AFAB) and trans masculine people at 32% and 30%, respectively—likely reflecting the invisibility of these groups in the HIV response. Importantly, the study found that having a transgender‐inclusive primary care provider was associated with lower odds of never having an HIV test—supporting the premise that training providers to be more culturally responsive and medically competent in the care of transgender patients may improve HIV outcomes in this population.

Research from multiple countries provides insights on HIV prevention strategies used by transgender people. Byrne et al. analysed data from 704 sexually active transgender and non‐binary people in Aotearoa/New Zealand [8]. Most study participants felt able to successfully negotiate the use of a protective barrier with a sexual partner. However, among participants attracted to men, self‐efficacy scores were lower for transgender men and non‐binary AFAB people than transgender women. Overall awareness of PrEP was high; however, knowledge about PrEP was higher among transgender men attracted to men than transgender women attracted to men. PrEP uptake was quite low overall at only 1%.

Aguayo‐Romero and colleagues used innovative latent class analyses to examine data from 958 transgender women across the Eastern and Southern United States [9]. They identified four latent classes of HIV awareness and prevention strategies used by site‐based and online participants: (1) “limited strategies—less sexually active” (15% and 9%, in site‐based and online, respectively), (2) “limited strategies—insertive sex” (16%/36%), (3) “limited strategies—receptive sex” (33%/37%) and (4) “multiple strategies—insertive and receptive sex” (36%/18%). The probability of reporting condomless sex was high in all classes. Membership in class 4 was characterized by PrEP use and may indicate the use of adaptive strategies in response to known HIV risk, while membership in class 3 indicated the use of a limited number of HIV prevention strategies in the face of heightened HIV vulnerability. Overall findings suggest prioritizing combination strategies, with a particular focus on HIV testing and PrEP, and indicate that future interventions may benefit from acknowledging the HIV prevention steps transgender women are already taking and offering tailored support to meet their goals.

A multicentre study (*n*=28 sites) assessing same‐day daily oral PrEP in Brazil, Mexico and Peru provided baseline data for Konda et al.’s study of factors associated with long‐term PrEP engagement and adherence among transgender women (*N*=494) [10]. Over 274.5 person‐years of follow‐up, 48% of transgender women demonstrated long‐term PrEP engagement, that is attendance at the 4‐week visit and two or more quarterly visits within a 52‐week period. PrEP adherence increased over follow‐up from 38% at the first visit to 53% at the final visit. In multivariable models, adherence was lower among participants who reported PrEP‐associated gastrointestinal symptoms, and PrEP engagement was higher among transgender women with 100% adherence at 4 weeks on PrEP. These findings highlight the importance of early adherence and addressing PrEP side effects to promote adherence and long‐term PrEP engagement.

We included four manuscripts describing innovative, transgender‐inclusive approaches to HIV service delivery. Two describe differentiated service delivery models in sub‐Saharan Africa [11, 12]. Mwango and colleagues describe a service‐delivery model in Zambia in which community health workers use a peer‐to‐peer community‐based approach in partnership with local transgender civil society organizations to reach transgender people for HIV testing, counselling and linkage to biomedical prevention or treatment [11]. This model reached more than 1800 transgender people over 8 months. Of the 424 transgender people who received HIV testing, 78% of those who were HIV negative (*n*=268) were initiated on PrEP and 97% of those who tested positive for HIV (*n*=78) were started on ART.

Bothma and colleagues provide programmatic data from dedicated differentiated healthcare centres for transgender people in four South African districts [12]. Unlike other sites in the country, these centres provide gender‐affirming hormone therapy and HIV services at a primary healthcare level. Over 18 months, they reached over 5000 transgender people via peer outreach and linked 62% of them to clinical services. Fourteen percent (*n*=687) of the 4829 who tested for HIV had a positive result and 91% of them initiated ART. However, only 28% (*n*=1165) of those who tested HIV negative accepted PrEP. The success of community‐based peer outreach for PrEP engagement in Zambia and South Africa provides evidence to support this strategy. The differences in PrEP uptake in the two programmes suggest the need for more data to understand what factors influence the success of community outreach for the engagement of transgender people in PrEP.

Doan et al. describe the implementation of participatory continuous quality improvement and Plan‐Do‐Study Act methods to increase PrEP uptake among transgender women in Vietnam [13]. This approach identified five key barriers and implemented corresponding solutions that included offering gender‐affirming care training, integrating gender‐affirming medical services (e.g. hormone therapy), implementing a transgender women‐led campaign addressing concerns about PrEP and hormone drug interactions, and the development of national HIV and transgender health guidelines. These initiatives significantly increased new PrEP enrolment and continuation among transgender women.

Rebchook et al. describe a variety of innovative interventions implemented to increase engagement in HIV care among transgender women of colour in nine sites across the United States, including housing linkages, legal services and peer support [14]. Heterogeneous, tailored, combination interventions were offered across the sites. Compared to baseline, each site significantly increased ART prescriptions and viral suppression over 12 and 24 months of follow‐up. Taken together, studies across southern Africa, Vietnam and the United States demonstrate that tailored interventions that address the specific needs of transgender people can be successful in increasing HIV prevention and care engagement.

The final three articles highlight the history of guideline development and emphasize the importance of best practices for engaging transgender people in HIV research. Given the significant geographic variability in the provision of care and treatment for transgender people across the globe, the World Health Organization (WHO) has a critical role to play in setting the fundamental standards of transgender‐inclusive HIV services. Macdonald and colleagues lay out the history of WHO's evolving role in facilitating quality, gender‐affirming, culturally responsive care for transgender people across the globe [15]. Articles led by Allison [16] and Klein [17] provide important insights on best practices for conducting research with transgender people. Allison and colleagues outline the importance of trauma‐informed approaches, given the well‐documented structural and interpersonal violence experienced by transgender people [16]. Klein and Golub lay out ethical challenges observed in research with transgender people and provide practical recommendations for the conduct of research that addresses these challenges [17].

While more data are clearly needed, especially among non‐binary people and transgender men, existing research provides an important road map for action. The articles in this supplement outline the unmet needs of transgender men, describe intervenable barriers and facilitators to HIV engagement and provide evidence of effective programmatic interventions across multiple continents. We hope the manuscripts in this supplement also make clear that the way forward must be led by and meaningfully engage transgender people. If we follow the science and engage communities, we have the necessary tools to end HIV inequities and advance the health and wellbeing of transgender people.

## COMPETING INTERESTS

The authors declare no competing interests.

## AUTHORS’ CONTRIBUTIONS

TP conceived and wrote the initial draft of the manuscript. SLR, NP and BG provided feedback, reviewed and edited the initial draft and approved the final version prior to submission.

## FUNDING

Publication of this open access special issue was supported by funding from Gilead Sciences, Merck & Co., Inc., and the National Institutes of Health via the HIV Prevention Trials Network. The authors alone are responsible for the views expressed in this issue. They do not necessarily represent the views, decisions or policies of the institutions with which they are affiliated nor any of the funding agencies.
